# Unlocking the Potential of Nutritional Approaches and Bioactive Compounds to Modulate Oxidative Stress and Inflammation in Systemic Arterial Hypertension

**DOI:** 10.1002/mnfr.70627

**Published:** 2026-10-12

**Authors:** Agnes Ciafrino, Denise Mafra, Marcia Ribeiro, Karen S. Coutinho‐Wolino, Thais Ionara, Victor Côrtes, Ronaldo A. O. C. Gismondi, Ludmila F. M. F. Cardozo

**Affiliations:** ^1^ Graduate Program in Cardiovascular Sciences Fluminense Federal University (UFF) Rio de Janeiro Brazil; ^2^ Graduate Program in Biological Sciences – Physiology Federal University of Rio de Janeiro (UFRJ) Rio de Janeiro Brazil; ^3^ Graduate Program in Nutrition Sciences Fluminense Federal University (UFF) Rio de Janeiro Brazil

**Keywords:** bioactive compounds, dietary patterns, hypertension, inflammation, oxidative stress

## Abstract

Systemic arterial hypertension (SAH) remains a leading global cause of premature death, significantly increasing the risk of cardiovascular, cerebrovascular, and renal diseases. While non‐modifiable factors, such as age and genetics, contribute to its development, lifestyle changes are crucial for prevention and management. Dietary modifications, such as reducing sodium intake, increasing potassium, and adopting a diet rich in fruits and vegetables, combined with regular physical activity, weight management, smoking cessation, and reduced alcohol intake are effective strategies for lowering blood pressure. Recent research has increasingly focused on bioactive compounds with antioxidant and anti‐inflammatory properties for the management of SAH. Substances such as polyphenols, curcumin, and other plant‐derived compounds, as well as live microorganisms such as probiotics, are being actively studied for their potential to improve endothelial function, modulate nitric oxide production, and reduce oxidative stress. This narrative review summarizes the current evidence on dietary interventions and bioactive compounds as complementary approaches to traditional treatments, examining their mechanisms of action.

## Graphical abstract

Polyphenols target oxidative stress and inflammation, improving endothelial function and reducing blood pressure in systemic arterial hypertension. Created by BioRender.com.

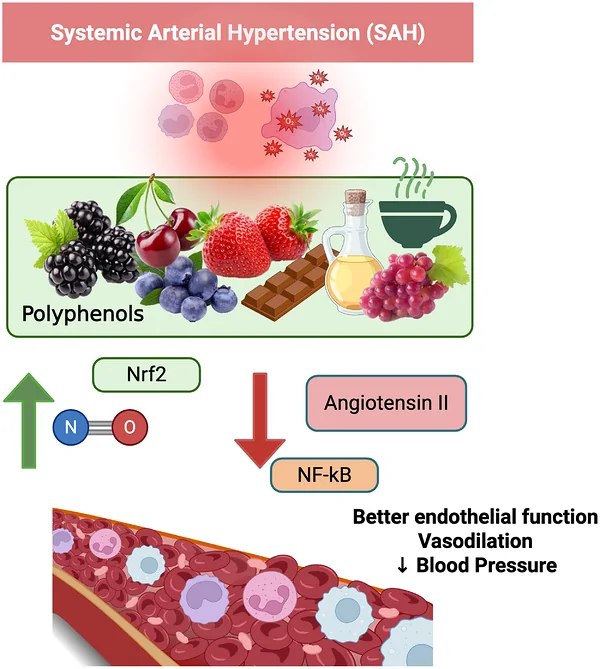

## 1 Introduction

According to the World Health Organization (WHO), the global prevalence of systemic arterial hypertension (SAH) has increased significantly, rising from 650 million cases in 1990 to 1.13 billion in 2019. Current estimates suggest that 1.28 billion adults aged 30–79 years globally are affected by hypertension [1]. The burden of hypertension is exceptionally high in low and middle‐income countries, where access to diagnosis and treatment is limited, highlighting the need for cost‐effective dietary strategies [1]. SAH is characterized by persistently elevated blood pressure (BP) (≥140 mmHg systolic or ≥ 90 mmHg diastolic) [2]. Recently, in 2024, the European Society of Cardiology (ESC) introduced a new BP category called "Elevated BP," defined as systolic BP (SBP) between 120 and 139 mmHg or diastolic BP (DBP) between 70 and 89 mmHg [2]. Hypertension causes target organ damage, increases the risk of cardiovascular, cerebrovascular, and renal diseases [3, 4], and remains one of the leading causes of premature death worldwide [1].

Although advanced age and genetic predisposition are non‐modifiable risk factors, lifestyle choices play a significant role in the development of hypertension. Major modifiable contributors include overweight and obesity, high sodium intake, excessive consumption of saturated and trans fats, insufficient fruit and vegetable intake, physical inactivity, smoking, and excessive alcohol consumption [5]. Given these modifiable factors, lifestyle interventions, such as adopting a healthy diet rich in fruits and vegetables, reducing salt intake with low‐sodium and high‐potassium salt, engaging in regular physical activity, losing weight, quitting smoking, and limiting alcohol intake, are effective strategies to lower blood pressure [4, 6, 7, 8].

High sodium consumption is a well‐established contributor to hypertension, primarily due to its impact on fluid retention and vascular resistance. Conversely, increasing dietary potassium intake has been shown to counteract sodium's effects, supporting vascular relaxation and reducing BP [2]. Moderate reductions in sodium intake, along with increased potassium intake, can result in significant BP decrease [9]. Well‐established dietary patterns such as the DASH (Dietary Approaches to Stop Hypertension) and Mediterranean diets have shown significant benefits in lowering BP due to their focus on plant‐based foods, mono, and polyunsaturated fats, and limited sodium intake [10, 11].

Pharmacological treatments remain essential for many patients, including diuretics, angiotensin‐converting enzyme (ACE) inhibitors, calcium channel blockers, and beta‐blockers, which are effective in controlling BP and reducing cardiovascular risks [5]. However, in recent years, significant attention has been given to bioactive compounds with antioxidant and anti‐inflammatory properties due to their potential in controlling hypertension [12]. Live microorganisms, such as probiotics, polyphenols, curcumin, and other plant‐derived substances, are being actively studied for their ability to enhance endothelial function, modulate nitric oxide (NO) production, and reduce oxidative stress [13, 14, 15]. These emerging treatments highlight a complementary approach, combining conventional therapies with nutraceuticals to achieve better blood pressure regulation and overall cardiovascular health.

Taking these factors into account, this review aims to synthesize current evidence on the role of dietary interventions and bioactive compounds in the prevention and management of hypertension, offering insights into their mechanisms of action and clinical relevance.

## 2 Oxidative Stress and Inflammation in Hypertension

SAH is associated with several conditions who can lead to the recruitment of innate immune cells, including macrophages, dendritic cells, and NK cells. In this environment, these cells change their transcriptional profiles, resulting in the activation of genes related to chemokine receptors and cytokine production. Consequently, these cells release pro‐inflammatory cytokines that attract additional immune cells, such as T cells and B cells, to the site of inflammation. This process further amplifies inflammation as these cells differentiate into effector cells (Th1 and Th17), which produce more pro‐inflammatory cytokines [16]. Specifically, macrophages, CD4+ and CD8+ T cells, along with interleukin‐6 (IL‐6), IL‐17A, IL‐1β, and interferon (IFN)‐γ, are suggested as mediators of hypertension development and end‐organ damage [17]. Indeed, individuals with SAH exhibit increased levels of several inflammatory mediators, including C‐reactive protein (CRP), IL‐6, and tumor necrosis factor‐alpha (TNF‐α) [18, 19]. These pro‐inflammatory cytokines seem to disrupt the function of the renin‐angiotensin‐aldosterone system by increasing both systemic and local production of angiotensin II, which causes vasoconstriction, which in turn promotes resistance to blood flow and ultimately culminates in elevated peripheral blood pressure endothelial dysfunction, and arterial stiffness, as well as the formation of atheromatous plaques [20]. This process triggers a vicious cycle, where elevated BP directly damages the vascular endothelium, worsening the hypertensive state [21].

The same factors that increase inflammation (increased levels of angiotensin II, sodium, catecholamines, and altered mechanical forces in the vascular wall) also trigger the cellular production of ROS, thereby contributing to oxidative stress [22]. The overproduction of ROS affects blood pressure through several mechanisms. Firstly, ROS can directly inhibit NO activity (a pivotal signaling molecule that regulates vascular blood flow, maintains vascular tone, and preserves endothelial integrity) and indirectly promote the activation of the PI3K/Akt‐MAPK pathway, which is related to the expression of redox genes that act by inhibiting the activity of endothelial nitric oxide synthase (eNOS), leading to endothelial dysfunction and, consequently, hypertension [23]. Additionally, under conditions of oxidative stress, NOS uncouples from its cofactor, generating harmful oxygen species that can harm vascular cells [24].

Moreover, NADPH oxidase (NOX) enzymes, considered the primary sources of ROS in the cardiovascular system, play an essential role in oxidative stress‐mediated hypertension. Specifically, NOX2 may promote BP elevation by inducing vascular dysfunction partly through paracrine signaling of vascular smooth muscle cells and activation of inflammatory pathways, including the nuclear factor‐κB (NF‐κB) pathway. Additionally, NOX5 appears to impair endothelium relaxation, and in vascular smooth muscle cells, this enzyme can induce vascular contractile responses and increase pulse pressure, favoring SAH [25]. NOX1 also appears to promote cardiac inflammation, impair endothelial relaxation, increase vascular remodeling and fibrosis, induce Angiotensin II‐mediated vascular hypertrophy, and consequently elevate BP [24].

High concentrations of ROS can also activate the nod‐like receptor pyrin domain‐containing 3 (NLRP3) inflammasome signaling pathway, a key component of the innate immune system and an important regulator of chronic inflammation. When activated, it stimulates caspase‐1, leading to the secretion of pro‐inflammatory cytokines, especially IL‐1β and IL‐18. These cytokines contribute to the polarization of T cells toward the production of excess IL‐17 and IFN‐γ. Elevated levels of these cytokines are frequently observed in individuals with hypertension and are well‐documented as favoring vascular dysfunction [17, 26]. Furthermore, in hypertension, NF‐κB is persistently overactivated in cells and tissues, and this process also stimulates the overexpression of NLRP3, leading to the consequent release of IL‐1β, which further aggravates the inflammatory environment [26]. It is essential to recognize that both oxidative stress and inflammation can mutually exacerbate each other, accelerating the progression of hypertension (Figure 1) [27].

**FIGURE 1:**
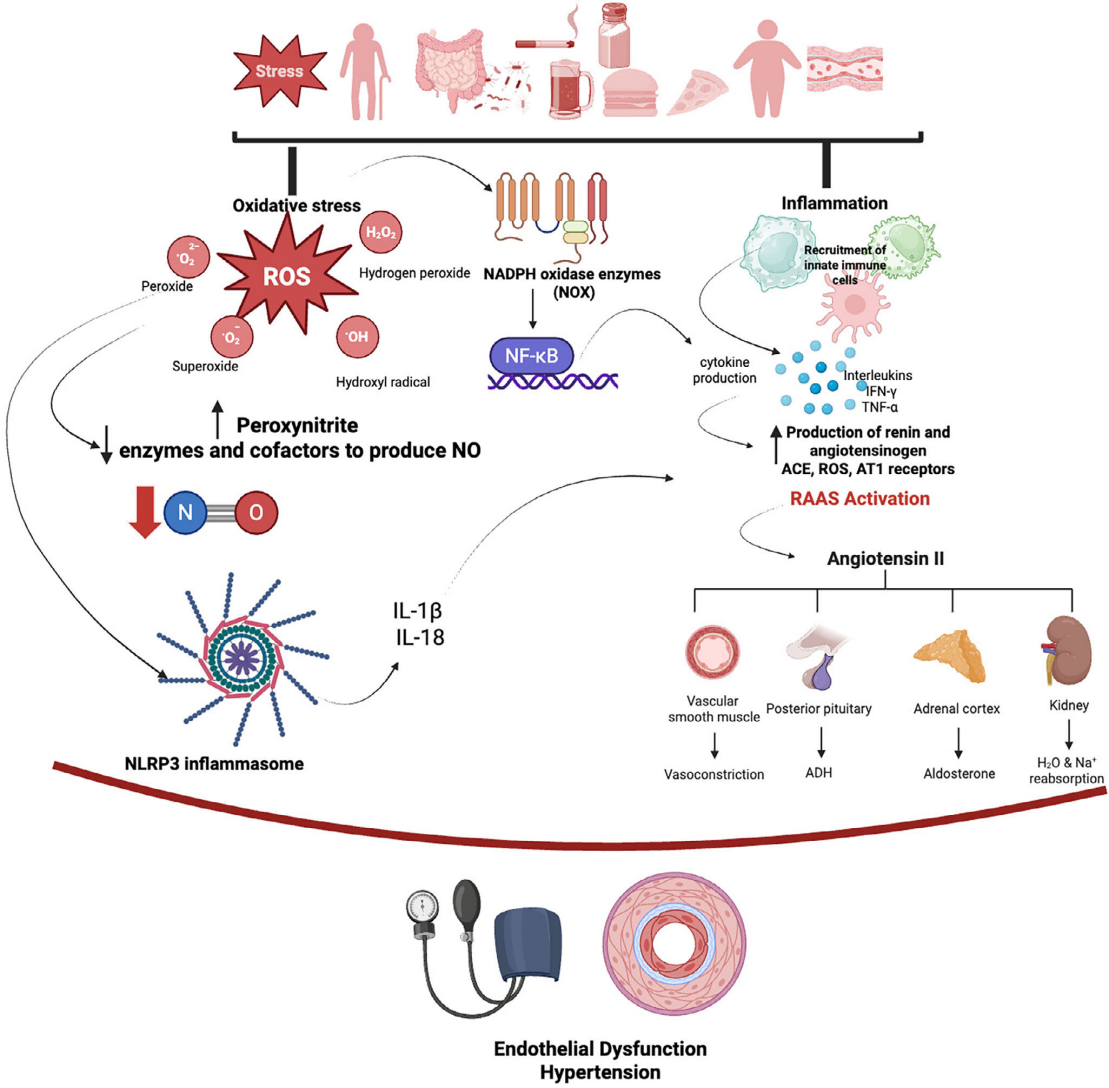
Inflammation and oxidative stress mediating systemic arterial hypertension. Chronic stress and metabolic disturbances promote the overproduction of ROS, including superoxide, hydrogen peroxide, and hydroxyl radicals, primarily via NADPH oxidase activation. Excess ROS interacts with NO, forming peroxynitrite, which impairs NO bioavailability and vascular relaxation. ROS also activates the NF‐κB signaling pathway in vascular and immune cells, triggering the transcription of pro‐inflammatory cytokines (e.g., IL‐1β, IL‐18) and activation of the NLRP3 inflammasome. Innate immune cells, such as macrophages and neutrophils, amplify inflammation through cytokine release and further ROS generation. These inflammatory mediators contribute to renin and angiotensinogen production, leading to RAAS activation, increased angiotensin II levels, vasoconstriction, aldosterone and vasopressin release, sodium and water retention, and elevated peripheral vascular resistance. Together, oxidative stress and inflammation drive endothelial dysfunction, ultimately contributing to the development and progression of hypertension. Abbreviations: IL, interleukin; NLRP3, Nucleotide‐binding domain, Leucine‐rich family, Pyrin‐3 domain; NF‐κB, Nuclear Factor‐κB; NO, nitric oxide; RAAS, Renin Angiotensin Aldosterone System; ROS, Reactive Oxygen Species.

The management of SAH can be divided into pharmacological and non‐pharmacological methods. Among non‐pharmacological strategies, dietary interventions play a crucial role in managing hypertension, often serving as a complementary approach to pharmacological strategy. Dietary patterns rich in fruits, vegetables, whole grains, legumes, and healthy fats, such as the DASH and Mediterranean diets, have consistently demonstrated antihypertensive effects. These benefits are primarily attributed to their high content of bioactive compounds, which possess anti‐inflammatory and antioxidant properties. Recent research has highlighted the role of specific bioactive compounds in modulating SAH progression and complications, thereby contributing to effective management of hypertension [28].

## 3 Nutritional Approaches

Among the most established measures, lifestyle changes stand out stand out, including recommendations for physical activity and dietary modifications [11, 29]. Meta‐analysis and clinical trials have already demonstrated a strong link between reducing salt intake, a well‐known recommendation for hypertensive patients, and lowering blood pressure [15, 30]. According to the ESC guidelines for adults with hypertension, it is recommended to limit total dietary sodium intake to approximately 2 g/day or less (equivalent to approximately 5 g, or about one teaspoon of salt per day). For the general population, a recommended sodium intake target is 2 to 4 g/day [2]. The American Heart Association (AHA) recommends that adults with high BP limit their sodium intake to no more than 1.5 g/day [31].

A meta‐analysis demonstrated that higher consumption of ultra‐processed foods (UPFs) is significantly associated with an increased risk of developing hypertension [32]. UPFs are highly palatable and typically consist of substances that contain added flavors, colorings, and other components such as additives and chemicals, as well as high levels of added sodium, sugar, and unhealthy fats, and have been associated with the development of hypertension [33]. In this context, Song et al.  [34] demonstrated, in a systematic review and dose‐response meta‐analysis, an improvement in the prevention and control of hypertension resulting from the reduction of pro‐inflammatory components in the diet.

### 3.1 Dietary Patterns

The Dietary Approaches to Stop Hypertension (DASH) diet, proposed in 1977 by the National Heart, Lung, and Blood Institute, aims to control the blood pressure of hypertensive patients through a dietary plan that includes food groups that have antihypertensive properties such as fruits, vegetables, whole grains, legumes, nuts, lean protein and low‐fat dairy products, in addition to reducing salt intake, added sugar and saturated fat [35, 36].

Nguyen et al.  [37] demonstrated that its implementation induces a hypotensive effect accompanied by hormonal and vascular adaptations. Moreover, it has a positive influence on cardiac parameters, including reductions in end‐diastolic and systolic volumes, and promotes improvements in left ventricular function [37]. DASH diet can also stimulate the inhibition of the ACE, directly affecting the renin‐angiotensin‐aldosterone system, which causes natriuresis and diuresis, resulting in BP reduction [38, 39]. Corroborating this mechanism, it is understood that the natriuretic action of the DASH diet can also be enhanced due to the potassium and calcium content present in the diet [40, 41]. Another vital role of the DASH diet is to attenuate inflammation through the action of antioxidant compounds such as polyphenols, including flavonoids. These bioactive compounds under pathophysiological conditions have already been associated with reductions in inflammatory markers, such as high‐sensitivity CRP (hs‐CRP), which may contribute to the improvement of subclinical cardiac injury through the attenuation of high‐sensitivity cardiac troponin I (hs‐cTnI) [42].

Although the DASH dietary pattern is the most studied in the world and several meta‐analyses and randomized clinical trials confirm its effects on the control of systolic and diastolic blood pressure [10, 43], a constant concern with the implementation of this pattern is to encompass cultural habits of different countries around the world and diverse ethnicities.

The Mediterranean diet (MedDiet) is based on a high consumption of antioxidant‐rich foods and anti‐inflammatory nutrients, in addition to fiber, poly‐ and monounsaturated omega‐3 fatty acids, and low levels of saturated and trans‐fats acids, as well as dietary cholesterol. As with the DASH diet, the MedDiet also recommends consuming whole grains, fish and seafood, low‐fat dairy products, poultry and eggs, fruits, vegetables, legumes, and nuts. However, the MedDiet emphasizes the incorporation of olive oil as the primary source of dietary fat and the moderate consumption of red wine during meals [39, 44].

Furthermore, the presence of fruits and vegetables, which are rich in vitamins, minerals, fiber, numerous bioactive compounds, and phytochemicals in both dietary patterns, significantly contributes to cardiovascular health. This is primarily achieved through endothelium‐dependent vasodilation and the inhibition of platelet aggregation [45]. Indeed, studies indicate that a diet rich in vegetables may be associated with a lower risk of mortality from ischemic heart disease, blood pressure control, and reduced need for medication [46, 47].

The strengths of the MedDiet are also attributed to the presence of olive oil, which provides monounsaturated fatty acids, vitamin E, and polyphenols, thereby increasing NO availability, promoting vasodilation, and enhancing endothelial function [48, 49]. Another critical component is red wine, which is rich in resveratrol, a polyphenol widely studied for its effects on cardiovascular health. Resveratrol is recognized for its antioxidant and anti‐inflammatory properties, as well as its role in preventing coronary heart disease [50]. Georgoulis et al.  [51] recently elucidated, in a prospective study involving 1,415 non‐hypertensive adults followed for 20 years, that adherence to the Mediterranean diet is longitudinally associated with a lower incidence of hypertension. Their findings, derived from the baseline MedDietScore, revealed that each one‐point increase in this score correlated with a 7% decrease in the 20‐year risk of developing hypertension [51].

Kong et al.  [52] investigated the relationship between overall fruit, vegetable, and legume intake, including their subgroups, and the risk of hypertension in adults aged 40 years or older over a 5.2‐year period. They observed general inverse trends with hypertension risk. However, the authors also noted that frequent consumption of pickled green leafy vegetables, such as pickled cabbage which are high in sodium, was positively associated with the risk of hypertension in postmenopausal women [52].

Among the dietary patterns observed by the authors are the DASH, Mediterranean (MedDiet), vegetarian (including pescetarian, lacto‐egg‐vegetarian, and vegan diets), low‐fat, low‐carbohydrate, and Paleolithic diets. However, at the end of the statement, the authors reinforce that the DASH, Mediterranean, pescetarian and vegetarian (egg, lacto, egg/lacto) dietary patterns were scored as the patterns that were most aligned with the recommendations of the Dietary Organization of the American Heart Association for the year 2021 [53].

Thus, seeking dietary patterns that, above all, promote health, respecting personal and cultural preferences and budgetary restrictions, is a therapeutic target encouraged in patients with SAH. Table 1 summarizes the main findings from the literature regarding dietary patterns in patients with hypertension.

**TABLE 1:** Studies involving dietary patterns in hypertensive patients.

References	Dietary patterns	Sample	Results
Chen et al. [108]	52% low‐sodium salt and DASH; normal salt and DASH	61 SAH patients with T2D: intervention group or control group (normal salt and DASH), for 8 weeks	**Intervention group**: ↓Sodium intake ↑Potassium intake ↓PP and MAP.
Ju et al. [109]	DASH dietary + one bag of oat bran (30 g/d, containing DF 8.9 g)	70 essential hypertensions: control group or intervention group, for 3 months.	↓the 24 h maximum HR ↓24 h average HR; 24 h maximum HR; average HR. During daytime; minimum HR during daytime; and maximum HR during daytime.
Vasei et al. [110]	DASH diet, based on vegetable or animal proteins	90 obese patients with metabolic syndrome: DASH diet, based on vegetable or animal proteins, for 8 weeks.	**In both groups**: ↓ Fasting plasma glucose, SBP and DBP, triglyceride, and weight.
Belanger et al. [111]	DASH diet *x* TAD	412 adults with high BP or SAH: 12 weeks of the DASH or TAD diet	**In both groups**: ↓ NT‐proBNP levels. **DASH diet**: ↓ High‐sensitivity cardiac troponin I and hs‐CRP.
Filipou et al. [11]	DASH diet with salt restriction *x* MedDiet with salt restriction	240 adults with high normal blood pressure or high‐grade SAH: medication; salt restriction; DASH diet + salt restriction or MedDiet + salt restriction.	**Compared to the medication group**: ↓ Office and 24‐h ambulatory SBP and DBP. ↓ SBP in the MedDiet group compared with all other study groups. Both diets were more effective in reducing BP compared with the salt‐restricted group.
Saslow et al. [112]	DASH *x* very low‐carbohydrate diet (VLCD)	94 adults with triple multimorbidity (SAH, prediabetes or T2D, and overweight or obesity) were randomized into one of two diet models.	**VLCD**: ↓ Mean SBP. Greater improvement in HbA1c and weight.
Zou et al. [113]	Low‐sodium salt applied to a Chinese modified DASH diet	61 older adults with SAH and T2D: low sodium salt or normal sodium salt group, for 8 weeks.	**Low sodium salt**: ↓ Ankle brachial index, brachial and ankle pulse wave conduction velocity. **The normal sodium salt**: ↓ Ankle brachial index
De Oliveira et al. [114]	DASH dietary guidance and dietary guidance plus encouragement to walk with a pedometer (DASHPED)	35 patients with T2D and uncontrolled SAH: DASH group or DASHPED group, for 16 weeks.	**DASHPED**: ↑ Physical activity of 1721 steps/day. **In both groups**: ↓ Weight, BMI, and waking diastolic pressures on ambulatory BP monitoring.
Sangouni et al. [115]	DASH diet versus healthy diet	60 individuals with metabolic syndrome: DASH diet or control diet group.	↓ Waist circumference, BMI, weight, SBP, DBP. ↓ Triglyceride, total cholesterol and LDL‐c.
Çapar, Yilmaz [116]	DASH diet and a salt‐free diet	60 patients with primary SAH: individualized DASH diet or salt‐free diet group, followed for 2 months.	↓SBP in salt‐free diet. **In both groups**: ↓ DBP.
Liebman et al. [117]	Whole‐food plant‐based diets (WFPBDs)	40 subjects with mild‐to‐moderate CKD and SAH but without diabetes or proteinuria, randomized to the 15‐day education program or the control group.	**WFPBDs group**: ↓ SBP. ↔ DBP. ↓ Potassium levels, body mass, total cholesterol, LDL‐c, and HDL‐c.

Abbreviations: BMI = body mass index, BP = blood pressure, DASH = dietary approaches to stop hypertension, DBP = diastolic blood pressure, HbA1c = glycated hemoglobin, HDL‐c = high‐density lipoprotein cholesterol, HR = heart rate, hs‐CRP = high sensitive C‐reactive protein, LDL‐c = low‐density lipoprotein cholesterol, MAP = mean arterial pressure, MedDiet = mediterranean diet, NT‐proBNP = N‐terminal pro‐B‐type natriuretic peptide, PP = pulse pressure, SAH = systemic arterial hypertension, SBP = systolic blood pressure, TAD = typical American diet.

### 3.2 Fruits and Vegetables as Sources of Polyphenols

Polyphenols are a diverse group of naturally occurring compounds found in fruits, vegetables, and seeds, known for their protective role against cellular damage and oxidative stress [54, 55]. Structurally composed of two or more phenol units, polyphenols are broadly categorized into flavonoids and non‐flavonoids, with flavonoids representing the most abundant subclass [55]. Dietary flavonoids, including anthocyanidins, flavonols, flavan‐3‐ols, flavones, flavanones, and isoflavones, are well‐known for their antioxidant, anti‐inflammatory, and vasoprotective properties. These bioactive molecules modulate enzymatic activity, increasing superoxide dismutase (SOD), catalase (CAT) and glutathione peroxidase (GPx), and interfere with cellular signaling pathways such as nuclear factor erythroid 2‐related factor 2 (Nrf2), thereby enhancing endothelial function, inhibiting platelet aggregation, reducing inflammation, and improving plasma lipid profiles, which collectively contribute to cardiovascular health [12]. The main non‐flavonoid classes include phenolic acids, lignans, tannins, and stilbenes, which also exhibit antioxidant and anti‐inflammatory activities [54].

Among flavonoids, compounds such as quercetin and catechin have demonstrated the ability to modulate key pathways, decreasing NF‐κB and increasing Nrf2, which contribute to reduced oxidative stress, suppression of pro‐inflammatory cytokines (e.g., TNF‐α, IL‐1, IL‐6), and increased expression of heme oxygenase‐1 (HO‐1) [12]. Quercetin, in particular, exhibits strong free radical‐scavenging activity by neutralizing ROS, including superoxide anion radical (O_2_•^–^) and hydrogen peroxide (H_2_O_2_) [54]. Furthermore, flavonoids exert antihypertensive effects by improving endothelial function, mainly through increased NO bioavailability, decreased ROS production, and activation of the PI3K/Akt/eNOS or AMPK signaling pathways [12]. Additionally, these compounds can influence vascular smooth muscle cells by promoting potassium channel activation and inhibiting calcium influx, leading to vascular relaxation and decreased vascular resistance [12, 56].

In particular, proanthocyanidins, a subclass of polyphenols, have been associated with reductions in systolic and diastolic blood pressure, as well as mean arterial pressure. These effects are attributed to increased NO production, improved endothelial function, and inhibition of ACE activity [13]. Similarly, observational studies across large U.S. and Chinese populations have consistently shown an inverse association between total dietary flavonoid or polyphenol intake and the risk of hypertension, with subclasses such as flavan‐3‐ols, anthocyanidins, and flavonols being particularly protective [57, 58, 59]. Beyond their vascular effects, anthocyanins exhibit antioxidant activity by activating the ERK/Nrf2 signaling pathway, which enhances the expression of HO‐1 and NQO1, thereby strengthening the body's antioxidant defense system and reducing oxidative stress [56]. Additionally, data from the PREDIMED trial indicated that moderate to high tomato consumption (>110 g/day, roughly equivalent to a large‐sized tomato), rich in the lycopene, was associated with significant reductions in DBP and lower risk of hypertension in older adults at high cardiovascular risk [60].

Lagou et al.  [61] conducted a meta‐analysis involving 5,205 participants to evaluate the effect of flavan‐3‐ol‐rich food, beverage, or supplement intake on BP and endothelial function. The authors included interventions with epicatechin, epigallocatechin‐gallate, cocoa products, tea, grape extract, and apples that supplied 586 mg of flavan‐3‐ols, and the results showed improvement in high blood pressure and endothelial function [61]. A meta‐analysis demonstrated that regular intake of tea, especially green tea, for more than 3 months can reduce SBP and DBP in individuals with high BP or hypertension [62]. Furthermore, another meta‐analysis demonstrated that green tea was also effective in reducing SBP and DBP in short‐term trials (6–13 weeks) [63].

Regarding the different types of teas, it was reported that green tea, in particular, contains the highest total content of phenolics and flavonoids such as epicatechin (EC), (‐)‐epigallocatechin, (‐)‐epicatechin‐3‐gallate, and (‐)‐epigallocatechin‐3‐gallate [64], in addition to having the most significant antioxidant power and free radical scavenging capacity [65]. The main pathways underlying the hypotensive effects attributed to regular tea intake include the maintenance of endothelial and vascular function, the improvement of blood flow by enhancing NO bioavailability, the suppression of the renin‐angiotensin‐aldosterone system, the scavenging of ROS, and the activation of antioxidant enzymes [64, 65, 66].

In addition to flavonoids, curcumin, a polyphenol found in turmeric, has shown promising antihypertensive potential [14], primarily attributed to its antioxidant and anti‐inflammatory properties, scavenging free radicals and enhancing endothelial function through upregulation of eNOS, which contributes to arterial vasodilation, reduction of arterial stiffness, and prevention of ventricular dysfunction [54, 67]. A recent systematic review and meta‐analysis revealed that curcumin or turmeric supplementation reduced SBP, DBP, and VCAM‐1 levels, while improving flow‐mediated dilation (FMD), suggesting enhanced endothelial function [14]. Another meta‐analysis, conducted by Karimi et al.  [68], included 1377 participants and demonstrated that curcumin consumption can improve DBP, provided it is consumed for at least 12 weeks [68]. Preclinical studies further showed that nanoemulsified curcumin had even greater efficacy, reducing BP and oxidative stress markers more effectively than conventional formulations. These effects are attributed to its antioxidant properties, including reduced lipid peroxidation and increased levels of antioxidant enzymes, such as glutathione, catalase, and superoxide dismutase [69].

Propolis, a natural resinous substance collected by bees rich in phenolic acids, and lignans, has also been associated with a significant reduction in SBP, although the effect on DBP was not statistically significant [70]. In addition, in vivo studies have demonstrated propolis antihypertensive effects by enhancing NO bioavailability, increasing antioxidant enzyme activity, suppressing the NF‐κB pathway, and reducing pro‐inflammatory cytokine levels [71]. Table 2 displays recent studies involving polyphenols in hypertension, and Figure 2 summarizes how the polyphenols modulate blood pressure.

**TABLE 2:** Clinical trial involving polyphenols in hypertension.

References	Study/sample	Intervention	Results
Marhuenda et al. [118]	RCT with pre‐hypertensive or type 1 hypertensive	500 mg of HS‐LC extracts (175 mg of HS + 325 mg of LC) or placebo for 84 days	↓ SBP and daytime SBP
Vors et al. [119]	RCT with pre‐hypertension	4 capsules/day of polyphenol extract (764 mg/day) + 2 g/day of L‐citrulline or placebo; for 6 weeks	↓ Ambulatory SBP in women
Sayec et al. [120]	RCT with prehypertensive	Encapsulated aronia berry extract (106 mg polyphenols) or placebo; for 12 weeks	↔ BP and endothelial function **Aronia extract**: ↓ Awake PWV and AIx
Sangouni et al. [121]	RCT with metabolic syndrome	Curcumin (200 mg) + placebo; Coenzyme Q10 (60 mg) + placebo; Curcumin (200 mg) + coenzyme Q10 (60 mg); double placebo group; for 12 weeks.	**Curcumin + Placebo**: ↓ TG, TC, LDL‐c ↑ HDL‐c **Curcumin+Q10**: ↓ TG ↔ SBP and DBP
Taladrid et al. [122]	RCT with high‐risk cardiovascular and healthy subjects	2 g day of Grape pomace seasoning or control, for 6 weeks.	↓ SBP, DBP, MAP
Tucci et al. [123]	RCT with mild hypertension	Acute effect of a portion of blueberry (250 g) versus a control product (C).	↑ RHI ↔ SBP and DBP

Abbreviations: AIx , augmentation index; BP , blood pressure; DBP , diastolic blood pressure; HDL‐c , high‐density lipoprotein cholesterol; HS‐LC , Hibiscus sabdariffa and Lippia citriodora; LDL‐c , low‐density lipoprotein cholesterol; MAP , mean arterial pressure; PWV , pulse wave velocity; RCT , randomized clinical trial; RHI , reactive hyperemia index; SBP , systolic blood pressure; TC , total cholesterol; TG , triglycerides.

**FIGURE 2:**
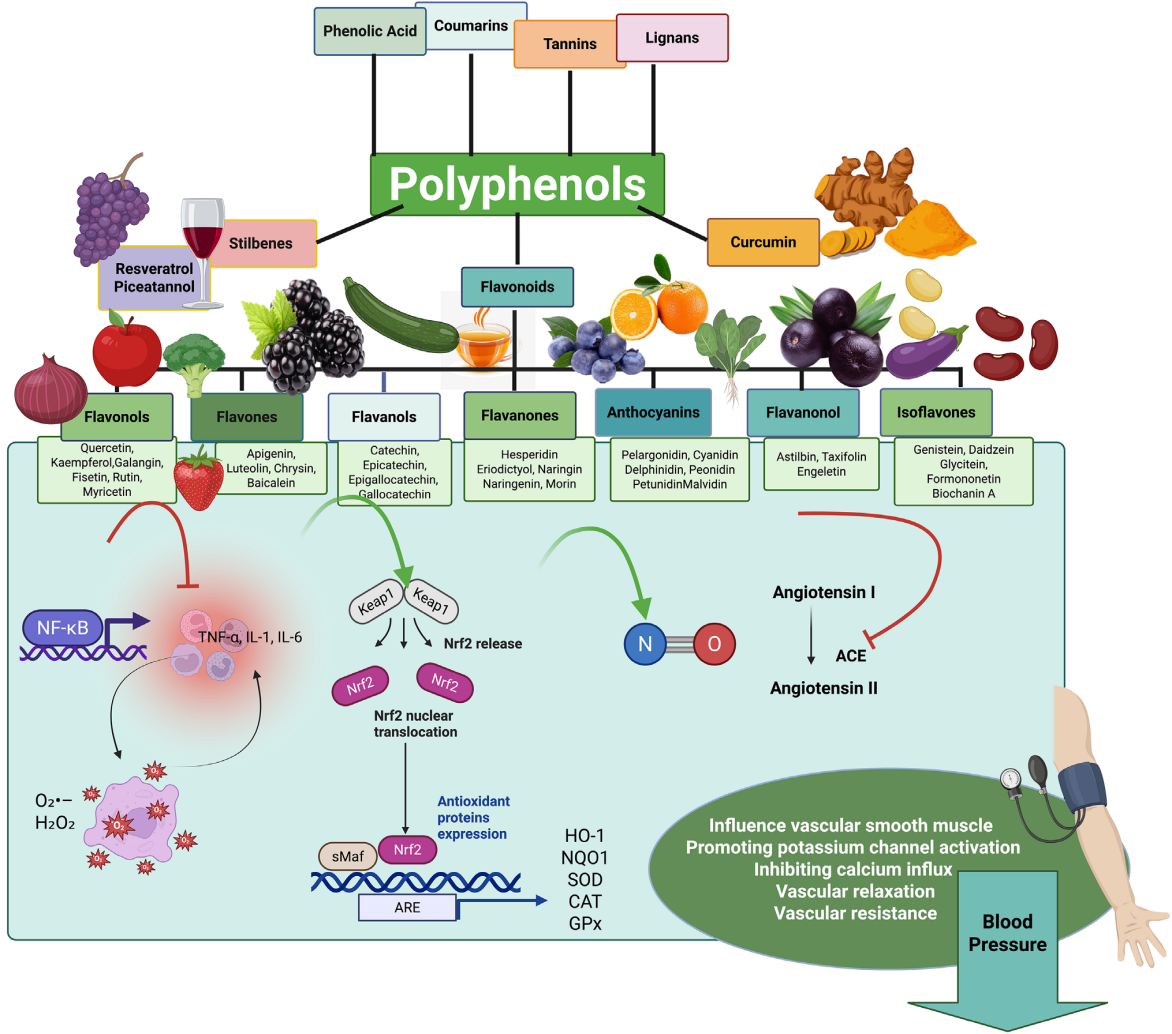
Mechanisms by which dietary polyphenols contribute to blood pressure regulation. Polyphenols exert antihypertensive effects through multiple pathways: (a) inhibition of NF‐κB activation, thereby reducing the production of pro‐inflammatory cytokines (IL‐1, IL‐6, TNF‐α); (b) activation of the Nrf2 pathway, leading to upregulation of antioxidant enzymes such as HO‐1, NQO1, SOD, CAT, and GPx; (c) enhancement of NO bioavailability, promoting endothelial function and vasodilation; and (d) inhibition of the renin‐angiotensin system, particularly ACE activity, reducing angiotensin II formation. Collectively, these actions contribute to improved vascular relaxation, reduced vascular resistance, and ultimately, blood pressure reduction.

### 3.3 Beetroot

Beetroot (*Beta vulgaris*), an edible taproot, has gained attention due to its health benefits, including antioxidant, anti‐inflammatory, anticarcinogenic, antidiabetic, hepatoprotective, and hypotensive properties [72]. It is rich in biologically active compounds such as betalains, flavonoids, polyphenols, and particularly inorganic nitrate (NO_3_^−^), alongside essential minerals like potassium, sodium, phosphorus, calcium, magnesium, copper, iron, zinc, and manganese, which collectively contribute to its therapeutic potential [72].

Dietary nitrate plays a key role in BP regulation. After ingestion, NO_3_^−^ is reduced to nitrite (NO_2_^−^) by oral anaerobic bacteria through the action of nitrate reductase. Nitrite is subsequently converted into NO in the acidic environment of the stomach and blood circulation under hypoxic conditions. NO, a potent vasodilator, rapidly diffuses into vascular smooth muscle cells, promoting relaxation via stimulation of cyclic guanosine monophosphate (cGMP) production and subsequent intracellular calcium extrusion. This cascade results in vasodilation, reduced peripheral resistance, and, consequently, lowered BP [73]. Beyond its endogenous synthesis from L‐arginine via nitric oxide synthase (NOS), the nitrate‐nitrite‐NO pathway offers a complementary route to increase NO availability, particularly relevant when endogenous NO production is impaired [73].

Similarly, a meta‐analysis by Benjamim et al.  [74] involving seven randomized controlled trials confirmed that nitrate‐rich BRJ intake led to a reduction in SBP. However, the impact on DBP was not statistically significant. Additional studies, such as those by Kapil et al.  [75] and Kerley et al.  [76], have shown that both short‐term (7 days) and longer‐term (4 weeks) BRJ supplementation enhances NO bioavailability and contributes to meaningful reductions in systolic and diastolic blood pressure. Furthermore, mechanistic studies indicate that NO‐induced increases in cGMP levels in vascular smooth muscle cells play a central role in promoting vasorelaxation, as demonstrated by Asgary et al.  [77] in interventions using raw and cooked beetroot. In line with these findings, Fejes et al.  [78] reported that 1 month of daily supplementation with nitrate‐rich BRJ (∼800 mg/day) in older adults with hypertension significantly increased plasma nitrite and nitrate levels, reduced oxLDL/NOx ratio and hs‐CRP concentrations, and improved redox status through a higher GSH/GSSG ratio, highlighting its antioxidant and anti‐inflammatory potential in this population.

Although the physiological functions of inorganic nitrate are recognized, moderate consumption should be recommended and the source should be evaluated, as excess may be involved in potentially harmful pathways. Under acidic gastric conditions, nitrite derived from nitrate can participate in the formation of N‐nitroso compounds in the presence of amine precursors, some of which have carcinogenic potential. Furthermore, heat treatments such as high‐temperature cooking, curing, or frying can promote the formation of nitrosamines from nitrate and nitrite. Despite this, evidence indicates that the health effects of nitrate consumption depend on the level of exposure, food source, food matrix and storage with risks generally higher associated with non‐vegetable sources such as processed meats but also processed food or contaminated water rather than fresh plant sources [79, 80, 81, 82].

### 3.4 Spirulina

Spirulina (*Arthrospira maxima* or *platensis*) is a blue‐green microalga rich in proteins, carbohydrates, essential fatty acids, carotenoids, γ‐linolenic acid, chlorophyll, and the notable phycocyanins. Recognized as a functional food, *Spirulina* has demonstrated significant antihypertensive potential, primarily attributed to its high content of phycocyanin and bioactive peptides with antioxidant and anti‐inflammatory properties, contributing to potential benefits in the management of cardiometabolic diseases, including hypertension [83, 84]. Spirulina acts to improve endothelial function by enhancing NO production and inhibiting NADPH oxidase activity [85]. Phycocyanin also exhibits anti‐inflammatory effects by modulating Toll‐like Receptor (TLR) and NF‐κB signaling pathways [86].

A systematic review demonstrated that *Spirulina maxima* supplementation has beneficial effects on BP, with its hypotensive effects primarily attributed to bioactive components such as phycocyanin and the tripeptide Ile‐Gln‐Pro [83]. These compounds act by increasing eNOS expression, enhancing NO production, and inhibiting ACE activity through the ACE‐I inhibitor peptide [84, 87]. The authors reported that a lower dose of 266 mg/day did not produce reductions in blood pressure, but doses above 2 g/day decreased SBP and DBP. Additionally, a dose of 4.5 g/day further reduced SBP and improved vascular health, with a reduction in the stiffness index [83]. Complementary to these findings, an in vivo study with hypertensive rat arteries demonstrated that the incubation with aqueous Spirulina extract enhanced vasodilation, associated with increased NO release, as well as upregulation of p‐Akt and HO‐1 expression [88].

Supporting these findings, a systematic review and meta‐analysis by Machowiec et al.  [89] found that Spirulina supplementation led to reductions in both SBP and DBP. Doses ranged from 1 to 8 g/day over periods of 2 to 12 weeks, demonstrating effectiveness particularly in hypertensive populations. Confirming these effects, Shiri et al.  [90] conducted a meta‐analysis of randomized clinical trials, assessed using the GRADE (Grading of Recommendations Assessment, Development and Evaluation) approach and the Cochrane assessment method were employed to evaluate the risk of bias. Spirulina supplementation was associated with a mean reduction of 4.41 mmHg in SBP and 2.84 mmHg in DBP [90].

A study by Szulińska et al.  [91] demonstrated reductions in IL‐6 levels and improvements in total antioxidant status in hypertensive and obese patients after Spirulina supplementation. These findings suggest that Spirulina supplementation may lower BP and improve vascular health, potentially through mechanisms involving enhanced NO bioavailability, antioxidant activity, and reduced systemic inflammation.

### 3.5 Probiotic

Changes in gut microbiota composition and function, known as gut dysbiosis, have been widely associated with SAH [92, 93]. Also, the proportion of the Firmicutes/Bacteroidetes phyla is increased. Regarding species, some bacteria are increased in individuals with SAH, such as *Eubacterium rectale*, *Klebsiella*, *Streptococcus*, and *Parabacteroides*. In contrast, others, especially those producing short‐chain fatty acids, are decreased, including *Roseburia* and *Bacillus freundii* [93]. Therapeutic strategies to reverse this hypertension‐associated gut dysbiosis are being explored. In this scenario, probiotics emerge as essential modulators of the disturbed microbiota [94].

From a pre‐clinical point of view, probiotics seem to have more consistent effects in improving BP, as shown by a range of studies in hypertensive rats [95, 96, 97, 98, 99]. The effect of metabolic endotoxemia has been adopted to explain the benefits of probiotics in improving hypertension‐associated oxidative stress. Probiotics can increase the number of butyrate‐producing bacteria, thereby improving gut barrier integrity. This process leads to lower translocation of bacterial endotoxin into the circulation and prevents activation of the Toll‐like receptor 4 (TLR4) pathway in the vasculature. The TLR4 pathway is well known for stimulating oxidative stress by increasing ROS, mainly NADPH oxidase O_2_, which subsequently reacts with NO, decreasing its bioavailability, favoring endothelial dysfunction, and affecting BP. Therefore, by improving gut integrity, probiotics can inhibit the TLR4 pathway in the vasculature and improve BP [97]. Moreover, probiotics may overregulate PPARβ and γ expression, which acts by inhibiting NF‐κB signaling and inflammatory response in the vascular wall, consequently improving hypertension‐associated inflammation [100].

### 3.6 Garlic

Garlic (*Allium sativum*) contains organosulfur compounds among its bioactive components. These organic compounds, which contain sulfur as a key structural element, have been linked to improvements in hypertension, and it is considered a potential adjuvant in the treatment of this cardiovascular condition [101, 102]. As a natural exogenous source of hydrogen sulfide (H_2_S), garlic releases this molecule, triggering various physiological effects. In this context, sulfur‐containing compounds in garlic promote vasodilation by enhancing H_2_S release, leading to antihypertensive effects [103]. H_2_S belongs to a class of molecules known as endogenous gasotransmitters, which, due to their solubility in both aqueous environments and membranes, play a role in vascular tone regulation. Reduced levels of H_2_S have been linked to CVDs, including hypertension, suggesting its crucial role in maintaining vascular health [103, 104]. It is well established that, over time, specific sulfur‐based constituents, such as allicin, undergo chemical transformations, resulting in the generation of novel bioactive compounds. One of the products of this modification is s‐allyl‐cysteine (SAC), which has antioxidant, anti‐inflammatory, and redox modulating action. SAC is present in high concentrations in aged black garlic (ABG), which makes this component potentially beneficial as it improves blood flow and is effective in the vasorelaxation process [105].

Garlic and Extract of Fermented Allium (EFA) have a mechanism of action similar to that of N‐acetylcysteine, which increases the bioavailability of NO. This vasodilator helps maintain blood pressure homeostasis. On the other hand, it also contains compounds capable of reducing the synthesis of ROS, which are responsible for NO consumption, such as O_2_‐. One of the vasodilation mechanisms that promotes BP reduction from garlic's bioactive compounds occurs when the enzyme cystathionine lyase (CSE) catalyzes the breakdown of sulfur compounds, such as those found in garlic, into hydrogen sulfide. H_2_S then acts as a signaling molecule that directly affects potassium channels in the blood vessels, inducing vasodilation and improving endothelial function. Therefore, garlic or EFA supplementation is potentially effective in reducing BP, especially in cases of mild hypertension [106].

The researchers observed that consumption of EFA tea significantly reduced SBP, and when administered in higher doses, it also reduced DBP levels. These results suggest that EFA supplements may serve as a valuable adjunct to conventional antihypertensive treatments [107]. The findings indicate that supplementation with various garlic‐derived bioactive compounds represents an effective non‐pharmacological strategy for supporting hypertension management (Table 3).

**TABLE 3:** Studies involving organic compounds from garlic and hypertensive patients.

References	Study/sample	Intervention	Results
Soleimani et al. [124]	RCT with 98 patients with nonalcoholic liver disease and diet‐controlled BP with or without antihypertensive medication.	400 mg garlic tablet (1.5 mg Allicin) or placebo, two times a day for 15 weeks.	↓ SBP, DBP, and MAP ↓ hs‐CRP
Fogacci et al. [125]	Non‐randomized, diet‐controlled clinical trial, 40 patients with high‐normal BP or hypertension I	DASH diet 2 weeks before intervention. The intervention group received 2 capsules of SelectSIEVE SlowBeat (140 mg black garlic + 60 mg of pomegranate per capsule), for 1 month.	↓ SBP and DBP
Mohammadi et al. [126]	Randomized, crossover, triple‐blind, placebo‐controlled clinical trial with hypertensive patients	5 g/day of dark chocolate (650 mg of microencapsulated FGE powder) or the placebo, for 6 weeks.	↔ BP, lipid profile, serum homocysteine, hs‐CRP, MDA and TAC
Houston et al. [127]	An open clinical trial with 12 patients with elevated blood pressure	2 capsules of Vascanox HP® tablets (242 mg of nitrate + SAC) once a day, for 4 weeks.	↑ Salivary NO_2_^−^, NO_3_^−^ ↓ SBP and DBP
Serrano et al. [102]	RCT with 77 participants with hypertension I	One tablet of AGB (250 mg of ABG extract) or placebo, for 12 weeks.	↓ SBP and DBP ↑ NO and antioxidant capacity ↓ ACE activity
Rahmatinia et al. [128]	RCT with 96 participants with prehypertension	Two capsules of freeze‐dried garlic extract daily or placebo, for 8 weeks.	↓ SBP, DBP, and MAP ↓ TG, LDL‐c, TC ↑ HDL‐c ↑ NO

Abbreviations: ABG , aged black garlic; ACE , angiotensin‐converting enzyme; BP , blood pressure; DASH Diet , dietary approaches to stop hypertension; DBP , diastolic blood pressure; FGE , fermented garlic extract; HDL‐c , high‐density lipoprotein cholesterol; hs‐CRP , high sensitive C‐reactive protein; LDL‐c , low‐density lipoprotein cholesterol; MAP , mean arterial pressure; MDA , malondialdehyde; NO , nitric oxide; NO^2−^ , nitrite; NO^3−^ , nitrate; RCT , randomized clinical trial; SAC , S‐alil‐L‐cisteína; SBP , systolic blood pressure; TAC , total antioxidant capacity; TC , total cholesterol; TG , triglycerides.

## 4 Conclusion

Despite advances in pharmacological management, hypertension remains a significant global public health concern, with persistent challenges in achieving optimal blood pressure control. The intricate interplay between oxidative stress, inflammation, and endothelial dysfunction plays a central role in the pathophysiology of hypertension, reinforcing the need for complementary strategies beyond conventional treatment. This narrative review critically highlights the growing body of evidence supporting dietary interventions and specific bioactive compounds, such as polyphenols, probiotics, curcumin, beetroot, garlic, and spirulina, as promising adjuncts in modulating key molecular pathways implicated in hypertension.

While numerous clinical and preclinical studies report antihypertensive effects of these compounds, primarily through enhancement of nitric oxide bioavailability, suppression of pro‐inflammatory cytokines, and attenuation of oxidative stress, several limitations persist. Notably, heterogeneity in study design, dosage, bioavailability, duration of interventions, and populations studied limits the generalizability and clinical translation of current findings. Furthermore, the efficacy of certain foods remains inconsistent, particularly in hypertensive populations, underscoring the urgent need for well‐designed randomized controlled trials with standardized protocols.

Moving forward, research should aim to establish mechanistic clarity, identify optimal dosing regimens, assess long‐term safety profiles, and investigate synergistic effects with pharmacological therapies. Additionally, tailoring nutritional strategies to individual genetic, microbiota, and lifestyle profiles may pave the way for precision nutrition in hypertension care. Until such evidence is robustly consolidated, dietary approaches should be considered supportive, rather than substitutive, tools in the multifactorial management of hypertension.

## Author Contributions

**Agnes Ciafrino**: conceptualization, writing – original draft, reviewing and editing. **Victor** **Côrtes, Ronaldo A. O. C. Gismondi**: reviewing and editing. **Marcia Ribeiro, Karen S. Coutinho‐Wolino and Thais Ionara**: writing – original draft, reviewing and editing. **Denise Mafra and Ludmila F.M.F. Cardozo**: conceptualization, reviewing and editing, supervision.

## Conflicts of Interest

The authors declare no conflicts of interest.

## Data Availability

Data sharing not applicable to this article as no datasets were generated or analysed during the current study.
